# Strategies to Overcome Intrinsic and Acquired Resistance to Chemoradiotherapy in Head and Neck Cancer

**DOI:** 10.3390/cells14010018

**Published:** 2024-12-27

**Authors:** Tycho de Bakker, Anouk Maes, Tatiana Dragan, Philippe Martinive, Sébastien Penninckx, Dirk Van Gestel

**Affiliations:** 1 Radiotherapy Department, Institut Jules Bordet, Université Libre de Bruxelles (ULB), 1070 Brussels, Belgiumsebastien.penninckx@hubruxelles.be (S.P.);; 2 Medical Physics Department, Institut Jules Bordet, Université Libre de Bruxelles (ULB), 1070 Brussels, Belgium

**Keywords:** chemoradiotherapy, head and neck, chemoresistance, radioresistance, acquired resistance, intrinsic resistance

## Abstract

Definitive chemoradiotherapy (CRT) is a cornerstone of treatment for locoregionally advanced head and neck cancer (HNC). Research is ongoing on how to improve the tumor response to treatment and limit normal tissue toxicity. A major limitation in that regard is the growing occurrence of intrinsic or acquired treatment resistance in advanced cases. In this review, we will discuss how overexpression of efflux pumps, perturbation of apoptosis-related factors, increased expression of antioxidants, glucose metabolism, metallotheionein expression, increased DNA repair, cancer stem cells, epithelial-mesenchymal transition, non-coding RNA and the tumour microenvironment contribute towards resistance of HNC to chemotherapy and/or radiotherapy. These mechanisms have been investigated for years and been exploited for therapeutic gain in resistant patients, paving the way to the development of new promising drugs. Since in vitro studies on resistance requires a suitable model, we will also summarize published techniques and treatment schedules that have been shown to generate acquired resistance to chemo- and/or radiotherapy that most closely mimics the clinical scenario.

## 1. Introduction

In 2021, the Belgian Cancer Registry reported 2788 new diagnoses, of which 1951 were male, making HNC still the fifth most common cancer diagnosed in men and the eighth most common in women in Belgium [1]. Additionally, 58% of patients on diagnosis exhibit stage III and IV cancers with a 5-year overall survival of only 10–50% [2,3]. When possible, these tumors are surgically resected, often followed by radiotherapy (RT) and even concomitant chemotherapy (CT) in case of positive margins or capsule rupture. Loco-regional unresectable cancer is treated with concomitant chemoradiotherapy [4] (CRT). Alternatively, EGFR inhibition with cetuximab can be administered, while the use of immune checkpoint inhibitors is not yet recommended. Despite the use of these aggressive treatments with significant toxicity, many tumours recur by intrinsic or acquired resistance, reducing the life expectancy of the patient [5]. In this context, there is a strong need for therapies that target specific resistance mechanisms in order to improve overall treatment outcomes.

In this review, we discuss the mechanisms by which HNC cells are/become resistant to CT and/or RT. Since in vitro investigation of resistance requires a suitable model, we also summarize known techniques and treatment schedules that generate acquired resistance to chemo- and/or radiotherapy. Finally, we summarize therapies that target these resistance mechanisms.

## 2. Mechanism of Resistance to Chemo- and/or Radiotherapy

**Figure 1:**
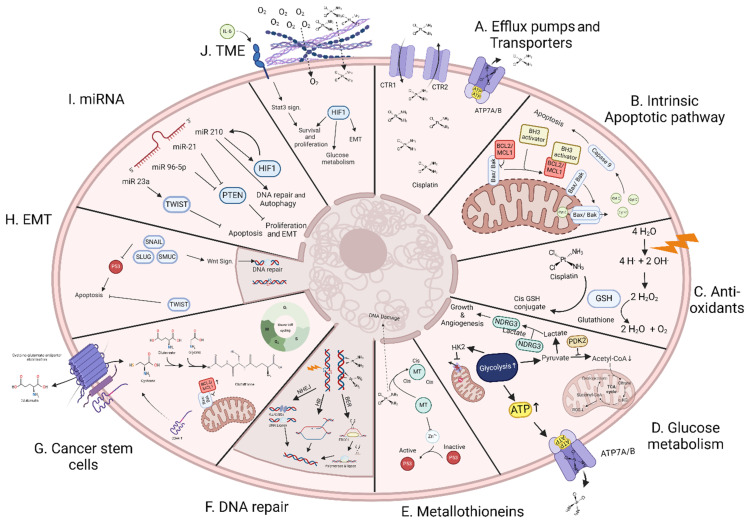
Global picture of the different resistance mechanisms: (A) Decreased intracellular cisplatin concentration caused by increased expression of cisplatin transporters. (B) Increased expression of anti-apoptotic proteins leads to a lack of cytochrome C release. (C) Increased antioxidant content results in the sequestration of cisplatin and degradation of toxic peroxides. (D) Metabolism change driven by the Warburg effect. (E) Metallothioneins chelate Zn^2+^ ions, which are essential for p53 functions, thereby preventing apoptosis through p53 activation and cisplatin sequestration. (F) Increased expression of DNA repair machinery components resulting in less persistent DNA damage and restoration of DNA integrity instead of apoptosis. (G) General properties of stem cells such as increased expression of glutathione and anti-apoptotic protein expression as well as general slower cell cycling. (H) Epithelial to mesenchymal transition (EMT)-induced expression of SNAIL, SLUG and SMUC inhibits p53, triggering Wnt signaling, which increases DNA repair. Moreover, TWIST activation inhibits apoptosis. (I) Several microRNAs, such as miR23a, miR96-5p, miR-21 and miR 210, confer resistance through many different signaling pathways such as PTEN, HIF1 and TWIST. (J) Chemotherapy and/or cytokines in the tumor microenvironment (TME), such as IL-6, influence the cellular characteristics of the cell, inducing its survival as well as the extracellular matrix preventing proper diffusion of both oxygen and cisplatin into the cell.

The efficacy of treatments vary from patient to patient due to different mutational profiles and tumor microenvironments (TME). Some patients exhibit intrinsic treatment resistance while others acquire resistance through exposure to their treatment, hence the apt terms intrinsic and acquired resistance, respectively. In this section, we will summarize the different possible resistance mechanisms that can occur for each of the monotherapies separately and when combined. These mechanisms will occur either by themselves or in combination. The combination of multiple mechanisms can contribute towards increased radioresistance compared to either mechanism alone (Figure 1).

Few studies have examined germline genetic variation as a potential marker of response to CRT in locally advanced HNC. Duran et al. evaluated the associations of 36 SNPs with response and survival of HNC to platinum-based CRT [6]. In addition to the study of individual associations with disease, performed for each SNP, a combined effect analysis was used to identify gene–gene interactions. One SNP of the *ABCB1* gene and three SNPs located in the *ERCC2* gene showed an association with response in the subset of HNC patients treated with definitive CRT [6]. These specific mutations are involved in mechanisms related to drug efflux pumps and DNA repair, respectively. When these resistance mechanisms are stimulated, they will induce resistance to CT and/or RT. We summarize a majority of the resistance mechanisms in a comprehensive manner below.

### 2.1. Efflux Pumps and Transporters

For a cell to survive, it must import or export many different solutes, either through osmosis or by using transport proteins such as pumps or channels. Some of these transport proteins can also transport molecules such as cisplatin and other chemotherapeutic agents. These include CTR1/2 and ATP7A/B, which are responsible for the import and export of excess copper ions. The efficacy of cisplatin is thus influenced by the balance between the expression of both types of transporters. When exporters like ATP7A/B are overexpressed, the cells are more resistant to cisplatin due to a reduced amount of intracellular cisplatin [7,8]. Simultaneously, transport of cisplatin via a copper transporter leads to degradation of this copper transporter, reducing the active influx and hence inducing resistance to cisplatin as a consequence, as passive diffusion is the only mechanism which still allows cisplatin entry [9]. Additionally, MDR1, also known as ABCB1, is an ATP-dependent efflux pump that is responsible for the efflux of many different substances, including several chemotherapeutic compounds [10]. Overexpression of this gene will also result in drug resistance [11].

### 2.2. Apoptotic Pathway

A large part of the apoptosis mechanism consists of proteins from the BCL-2 family, which contains both pro-survival proteins (BCL-2, BCL-xL, MCL-1, BCL-W, BFL1), effector proteins (BAK, BAX, BOK), BH-3 only activator proteins (BIM, BID, PUMA) and sensitizer proteins (NOXA, BAD, BMF, BIK, Hrk) [12]. Overexpression of the pro-survival BCL2 proteins in patients results in resistance to CRT [13]. Similarly, MCL-1, a frequently overexpressed anti-apoptotic protein, has recently become a major target in treating cancers [14]. Under normal circumstances, MCL-1 prevents the oligomerisation of effector molecules. However, upon binding of an MCL-1 inhibitor, the effector monomers are released and oligomerize into pores that release cytochrome C, which in turn activates caspases, leading to apoptosis. ANO1 is also involved in the regulation of MCL1 expression. In 30% of HNC cases, *ANO1* is amplified and overexpressed, resulting in resistance [15]. Outside of HNC, this gene has been implicated in therapy resistance of multiple different cancer types [16,17,18]. In addition to its effect on MCL1, ANO1 induces downregulation of p27, a cell cycle checkpoint protein found to be distributed in the cytoplasm, where it cannot exert its function. This results in unchecked cell cycle progression and subsequent failure to undergo apoptosis when required [15]. Similarly, CREB5 is also involved in the downregulation of the apoptosis mechanism through the upregulation of mitochondrial TOP1, which will upregulate the expression of BCL-2 and BCL-XL and inhibit the expression of Bax and cytochrome c [19]. AATF is often overexpressed in HNSCC, where it is associated with an increased STAT3/survivin pathway signaling and caspase 9 inhibition. This prevents apoptosis of the cell and confers resistance to cisplatin [20].

### 2.3. Antioxidant Defenses

Oxidative stress plays an important role in cancer development and therapy response, either by inducing cell death or as a secondary messenger. Mammals have developed a range of antioxidant defenses to regulate ROS levels and safeguard essential biomolecules from their harmful effects. These defenses include small endogenous molecules, like reduced glutathione (GSH), which can directly react with reactive oxygen species (ROS), as well as complex enzymes capable of repairing the modifications/damages caused by ROS. Overexpression of HSP25 is associated with an increase in GSH, which in turn scavenges a larger amount of radiation-generated ROS. This decreases the amount of indirect DNA damage produced by ionizing radiation, reducing the efficacy of RT treatments [21]. In addition, this HSP25 overexpression reduces the response to several chemotherapeutic drugs such as cisplatin. Besides binding DNA, cisplatin also binds sulfhydryl groups present on antioxidant molecules such as GSH. Interestingly, studies report that cisplatin binds GSH at the same reaction rate as it would bind to DNA [22], sequestering the cisplatin in the cytoplasm and preventing it from binding DNA. However, the binding of GSH prevents it acting as an antioxidant. This, in turn, leads to an imbalance in the redox system and thus to cell stress, which can ultimately contribute to cell death.

In a similar vein, the further reduction of oxidized GSH by recycling proteins such as the thioredoxin (Trx) system also contributes towards a more favorable ROS balance, by more quickly and more frequently reducing GSH, which in turn can act on any ROS that might be present. Additionally, these enzymes may react with already damaged proteins by reducing the oxidized residue on the target protein whenever possible, once again reducing the damaging capabilities of ROS [23]. While there is only limited research that has been performed on a combination of RT or CT in combination with any Trx inhibitors in HNC, combining auranofin and buthionine sulfoximine, which are Trx and GSH inhibitors, respectively, does reduce the clonogenic capacity of resistant cells [24]. Glutathione peroxidase, another GSH recycling protein, is also heavily linked to therapy resistance. Once again, when inhibiting this protein, the cells have less reduced GSH and thus will accumulate ROS, causing damage and potentially cell death [25].

Under oxidative stress, Keap1 dissociates from NRF2. This allows NRF2 to bind the ARE promoter construct, resulting in the expression of genes responsible for cellular redox homeostasis such as glutathione reductase, superoxide dismutase, thioredoxin reductase and catalase, among others [26], and xenobiotic detoxification. Additionally, these genes are responsible for the induction of ferroptosis when the ROS levels become too high [27]. Thus, overexpression of NRF2 results in a reduction of ROS and inhibition of ferroptosis, thereby conferring resistance to CRT [28,29].

### 2.4. Glucose Metabolism

One of the hallmarks of cancer, known for years as the Warburg effect, is an altered expression of proteins enabling tumor cells to use the anaerobic glycolysis pathway for energy production even under normoxic conditions. This phenomenon enables tumors to increase their production of ATP and leads to multiple mechanisms contributing to chemoresistance [30]. Hexokinase (HK) plays a major role in the induction of Warburg effect. The function of HK is to catalyze the first irreversible step of glycolysis during which glucose is phosphorylated to glucose-6-phosphate. Interestingly, the isoform HK2 is the only isoform of HK documented to be upregulated in tumors, especially in HNC. Research results show it decreases pyruvate dehydrogenase complex activity, rerouting the metabolic pathway to promote the Warburg effect [31]. Moreover, it inhibits apoptosis by interacting with anion channel proteins in the mitochondrial membrane, preventing the release of cytochrome C and thus causing resistance to CT [32,33]. The PI3K signaling pathway, which is overexpressed in many cancers, also upregulates HK2, thereby contributing to resistance. The resulting higher ATP content increases binding to the ATP cassettes of efflux pumps, increasing efflux of CT [34].

PKM2 is involved in the final step of pyruvate formation and produces excessive amounts of lactate in cancer cells. This lactate, in turn, binds NDRG3 and causes the activation of hypoxia-related pathways independent of HIF-1. NDRG3 lactate accumulates and phosphorylates c-Raf, resulting in growth and angiogenesis through ERK signaling [35]. Pyruvate dehydrogenase kinase 2 (PDK2) phosphorylates and inhibits the pyruvate dehydrogenase complex (PDC), which metabolizes pyruvate into acetyl-CoA. This lack of acetyl-CoA prevents the products of the tricarboxylic acid (TCA) cycle from entering the mitochondrial glycose oxidation and the electron transport chain, thereby decreasing the ROS production [36].

Due to their reliance on the Warburg effect, HNSCCs have been found to overexpress glucose transporter 1 (GLUT1), allowing for increased uptake of glucose [37]. Additionally, overexpression of GLUT1 has been associated with chemoresistance in other cancers [38]. Knockdown of GLUT1 with GLUT1-shRNA, as well as inhibition of GLUT1 by anti-GLUT1 antibody, sensitizes HNSCC to cisplatin, providing another promising treatment strategy for chemoresistant HNSCC [39].

### 2.5. Metallothioneins

The expression of metallothioneins (MTs) has been shown to be implicated in the resistance of several cancers to CT and RT [7,40,41]. MTs are cysteine-rich (30% of amino acids) proteins that chelate various metal ions involved in homeostasis. Additionally, MTs protect against DNA damage and oxidative stress [42]. While their chelating properties make MTs protective to the cell, once the cell has become oncogenic, some properties can contribute towards cancer progression and resistance to both chemo- as well as radiotherapy. One hypothesis is that MTs chelate zinc ions, which are essential for proper p53 function, and thus preventing proper apoptosis after treatment with either CT or RT. Additionally, MTs could chelate platinum-based therapeutic molecules themselves and reduce their intracellular concentration, hence contributing towards resistance [43,44].

### 2.6. DNA Damage Repair

Cancer cells tend to upregulate DNA repair mechanisms to prevent cell death [45]. Damage caused by chemotherapeutic agents such as cisplatin are often repaired by nucleotide excision repair (NER). In several cancers, upregulation of NER-related genes results in increased resistance to platinum-based chemotherapeutics [46]. The XPF protein, frequently upregulated in HNC, results in increased NER and thus chemoresistance [47]. RT causes many forms of DNA damage, of which double strands breaks (DSBs) are the most lethal and responsible for the expected therapeutic effect. To cope with these DSBs, cells use two main repair mechanisms, either non-homologous end joining (NHEJ) or homologous recombination (HR), to repair lesions [48].

Many different proteins involved in DNA repair mechanisms have been identified as being upregulated in tumor cells. Among those is ACTL6A, a subunit of chromatin remodeling complexes, which has been shown not only to drive the development of SCCs [49] but also to induce chemoresistance when the gene is amplified [50]. Overexpression of ACTL6A results in increased BAF saturation and reduced chromatin folding [49,51], limiting the number of DNA adducts induced by cisplatin [50]. Another protein, RPA1, a heterotrimeric single-stranded DNA-binding protein complex involved in DNA replication, recombination and repair, was identified to confer radioresistance when upregulated. Curiously, spontaneous DNA damage occurring throughout replication of the cell was also increased, demonstrating the involvement of RPA1 in cell cycle progression [52]. Other markers such as XRCC1, DNA polymerase β, PNKP and PARP-1, have been shown to be upregulated in HPV+ HNSCC, resulting in increased base excision repair and single-strand break repair. PARP-1 inhibition by olaparib restores radiosensitivity to a greater extent in HPV- rather than in HPV+ HNSCC [53]. In 2016, Umemura and Ihkoshi showed that overexpression of CD44 on cancer cells induces resistance via enhanced DNA repair. Inhibitors of CD44 expression reduce the amount of DNA repair via CHK1 phosphorylation [54] and increase cell cycle arrest, triggering apoptosis [54].

### 2.7. Cancer Stem Cells

Among all cell populations in a tumor, cancer stem cells (CSCs) are capable of regenerating a tumor when all the other cells in the tumor are destroyed. In most cases, these often dormant CSCs survive the treatment due to various intrinsic factors such as increased DNA repair capacity, enhanced management of both reactive oxygen and reactive nitrogen species, and by disabling apoptotic pathways [55,56]. CSCs also overexpress aldehyde dehydrogenase, which detoxifies aldehydes generated by CT [57]. One of the most prominent markers to identify CSCs is CD44 [58], which is the hyaluronic acid receptor and a co-receptor for chemo and cytokines, which then result in intracellular signaling, resulting in the expression of genes involved in cellular behavior. CD44 activation stabilizes the cystine–glutamate transporter [59], which provides the cell with cystine used in the glutathione synthesis, hence avoiding oxidative stress. Thus, CD44 expressed on cancer cells (including HNSCC) leads to the resistance and survival of these cells [60]. Recent evidence suggests that this marker, and more specifically the CD44v subtype, is also involved in metastasis process in addition to stemness.

Among the variety of cellular perturbations caused by chemo and radiotherapy, ER stress is caused by both. When the unfolded protein response is active for a prolonged period of time, it can induce apoptosis [61]. Stem cells, however, have upregulated GRP78, a chaperone protein present on the ER, which will release upon the detection of misfolded proteins. Subsequent refolding of said proteins reduces ER stress and thus confers therapy resistance in those cells [62]. In cancer cells, this protein is not only present on the ER but also expressed on the cell membrane, resulting in more stemness-related phenotypes and increased refolding of potentially misfolded proteins [63,64].

Several other proteins and signaling pathways are upregulated in cells presenting with stemness characteristics. These may subsequently influence the treatment response of the cell to chemo- and/or radiotherapy. Another example involved in the self-renewal of CSCs is the Wnt/β-catenin pathway, a pathway which is normally only active during embryonic development [65]. In CSCs, however, it is also active, inducing both stemness and therapy resistance through the activation of PI3K signaling and YAP/TAZ- mediated transcription [66,67]. Additionally, Wnt/β-catenin signaling increases anti-apoptotic pathways and induction of EMT.

CSCs have intrinsic properties related to their stemness that also contribute to therapy resistance. CSCs are considered to be quiescent, meaning that they cycle very slowly compared to other cancer cells or they are even in the G0 phase of the cell cycle. Therefore, they are more resistant to treatments that target proliferating cells such as RT and CT [68,69]. In addition, the balance of pro- and anti-apoptotic signaling, which we described in an earlier section, is different in CSCs compared to normal cancer cells. In CSCs, the amount of anti-apoptotic proteins is high, making the increase in pro-apoptotic proteins insufficient to result in apoptosis [70,71]. DNA damage response and antioxidant defenses are also upregulated on CSCs and result in enhanced resistance as described above [72].

### 2.8. Epithelial-Mesenchymal Transition

Epithelial–mesenchymal transition (EMT) is primarily activated during embryonic development in order to initiate development of the internal organs. During this process, the transitioning epithelial cells reside in a specific cellular niche. For the transition to take place, the cells need to detach from the basement membrane, leaving their place to be filled up by the remaining cells. At this point, the cells undergo phenotypic changes that are more mesenchymal-like, allowing for the cell to invade tissues and subsequently begin to form an internal organ [73]. Most healthy cells have disabled EMT and will irreversibly remain epithelial cells. In some organs, EMT can be induced to enable repair and fibrosis. In the tumor environment, EMT can be induced, resulting in loss of adhesion and a more mesenchymal-like phenotype. Once these cells detach from the tumor, they can enter the bloodstream or lymph nodes to spread out throughout the body and find a metastatic niche where they can develop into a metastatic tumor [74]. The changes in gene expression of mesenchymal cells also confer increased resistance to therapy compared to their epithelial counterparts. Irradiated cells are often induced to undergo EMT in response to radiation [75,76].

Among the activated genes are *SNAIL*, *SLUG* and *SMUC*, members of the SLUG superfamily. These induce a change in the cells that makes them more stem-like, thereby also making them more resistant to chemo- and radiotherapy, as mentioned previously [77]. In addition, activation of SNAIL and SLUG will antagonize the function of p53 and thus prevent apoptosis [77]. For example, radiation-induced ERK1/2 activation inactivates GSK3B, resulting in the upregulation of *SNAIL* [78]. Similarly, the Wnt signaling pathway is involved in EMT and also induces therapy resistance by upregulating of DNA damage repair and facilitating transcriptional plasticity [79,80]. A similar effect is observed for NFkB signaling, which is also involved in EMT. Nf-kB signaling prevents ubiquitination and degradation of SNAIL [80]. In the case of HNSCC, this is significant, since a modulator of NF-kB is p53, a very frequently mutated gene in HNSCC [81]. Cells undergoing EMT also have increased DNA repair capacity. When treating the HNSCC cell line SCC25 with EMT-inducing conditioned medium, a significant increase in ERCC1 expression occurs, which is responsible for increased DNA repair and expression of the anti-apoptotic marker survivin, once again resulting in radioresistance [82]. In the same experiment, another cell line, Detroit-562, showed the same radioresistant properties while not increasing the markers known for EMT. The authors attributed this effect to epithelial–mesenchymal crosstalk (EMC). EMC occurs when epithelial cells interact with the surrounding stroma [82]. A final marker attributed to EMT is TWIST, which has been shown to allow the accumulation of DNA damage without induction of apoptosis.

### 2.9. Non-Coding RNA

miRNA and shRNA affect many proteins, which cause resistance through many different mechanisms. They can be divided into five main groups: rRNA and tRNA, which are involved in translation (also called housekeeping RNA, while the others are sometimes called regulatory ncRNAs), snRNA in DNA splicing, snoRNA in modifications of other RNA molecules, and siRNA that are involved in gene regulation and silencing. The fifth group of ncRNA (tsRNA, circRNA and lncRNA) has a diverse array of functions [83].

Many of the miRNAs, shRNAs and siRNAs are involved in the resistance of cancer cells to treatment. Some squamous carcinomas of the tongue are known to have upregulated miRNA miR-23a, which in turn upregulates TWIST expression. It has been shown that this interaction increases the IC50 value for cisplatin as well as the signaling of the JNK pathway. Similarly, these phenomena disappear when miR-23a is knocked down [84]. miRNA-96-5p confers resistance not only to CT but also to RT by knocking down PTEN and increasing the capacity of cell migration [85,86]. Its overexpression is linked to cases containing mutated *TP53*. Another miRNA that confers chemoresistance is miR-21 [87]. It is normally activated by hypoxia and cytokines. miR-21 targets PTEN as well as TPM1 and PDCD4. Essentially, therapy resistance can be caused by the decrease in PTEN expression and thus also downstream signaling of PTEN. Hence, it is not necessarily the miRNA itself that confers therapy resistance but rather the specific effect of the miRNAs on signaling pathways that affect the therapy sensitivity and subsequently also the resistance [88]. Another miRNA causing therapy resistance, called miR210 [89], is induced by HIF-1α. miR-210 controls its target genes and results in increased DNA repair, autophagy and apoptosis inhibition. miR-210 also increases expression of HIF-1α, resulting in a positive feedback loop [90]. miR-630 is involved in the NRF2-GPX2 axis, where it induces upregulation of the antioxidant system, resulting in reduced ROS. In addition, the amount of γ-H2AX present in irradiated cells expressing miR-630 is reduced, indicating reduced DNA damage [91]. All aforementioned miRNAs are part of one of five resistance mechanisms involved in DNA damage detection and cell cycle arrest, DNA repair, cell apoptosis and EGFR signaling, which is specifically important for HNSCC due to its overexpression and EMT.

Although its effect in HNSCC is unknown, miR-197-3p has been shown to downregulate ZIK1, which regulates survival [92] and is downregulated in multiple tumors including HNSCC [92,93,94]. Exosomes enriched with miR-197-3p can radiosensitize HNSCC [95].

### 2.10. Tumour Microenvironment

While the tumor microenvironment (TME) has been extensively investigated for modulating the immune response against the tumour and the related response to immunotherapy, some factors in the TME can also contribute to the resistance to more conventional treatments such as CT and RT.

When the tumor increases in size, improper vascularization of the tumor results in decreased amounts of oxygen reaching certain regions of the tumor [96]. The oxygen concentration in these tumors can become as low as 1.3% (hypoxia), in contrast to the 5.3–6.7% (normoxia) of normal tissues [97]. While the direct effect of RT on DNA is not affected by the oxic state of the cell, the indirect effect via the generation of oxygen radicals is. The direct damage is often more difficult to repair [98]. When the cells lack sufficient oxygen, indirect damage is greatly reduced [99]. Since we estimate that 70% of DNA damage produced by X-ray is indirect, hypoxia allows a lower generation of DNA damage per dose delivered, resulting in cell survival and thus resistance. Additionally, hypoxic cells cycle slower compared to normoxic cells. This gives them extra time to repair any damage caused by RT and/or CT [100]. Additionally, the lack of oxygen will push the cells towards an anaerobic glycolysis, inducing increased lactate production and resulting in resistance, as mentioned previously [36].

Additionally, hypoxia induces the dimerization of HIF1 α and β subunits, which results in the expression of genes under the control of hypoxia response element. HIF1 also induces many of the components of the aforementioned mechanisms such as EMT, glucose metabolism and general survival and self-renewal pathways.

Moreover, improper vascularization can prevent proper delivery of chemotherapeutics to the tumor [101]. Depending on the density of the extracellular matrix, chemotherapeutic molecules could be hindered in their diffusion towards the cell and even physically be blocked [102]. This may be due to the deposition of large amounts of fibers such as collagen [103], laminin [104,105], fibronectin [104,106,107] and periostin [108].

Certain cytokines present in the TME are also involved in the induction of survival and EMT pathways, hence leading to resistance. IL-6 has been shown to confer resistance in erlotinib-resistant cells by increasing STAT3 signaling in resistant clones compared to their parental cell lines [109].

## 3. Recent Advances in Sensitizing HNSCC Cells to CRT

The unravelling of new resistance mechanisms provides new targets that are overexpressed or mutated. Targeting these proteins may constitute an opportunity to improve the outcome of patients who present with resistance. In the next section, we summarize the different treatments that have been developed to target a specific resistance mechanism.

### 3.1. Targeting DNA Damage Response

Ataxia-telangiectasia mutated kinase (ATM) and ataxia-telangiectasia and Rad3-related kinase (ATR) are the master transducers of DNA damage response [110,111]. ATR and ATM respectively phosphorylate and activate checkpoint kinases (Chk1 and Chk2), which induce cell cycle arrest and recruitment of DNA repair proteins.

AZD6738 (ceralasertib), an inhibitor of ATR, disrupts this pathway by preventing CHK1 phosphorylation [112,113]. When used in combination with cisplatin, AZD6738 enhances sensitivity to the drug both in vitro and in vivo [114], as demonstrated by increased DNA damage and cell death. Interestingly, it also has been reported to radiosensitize HNSCC in vitro and in vivo [115,116], paving the way to one clinical trial in HNSCC (NCT03022409), although no results have been published yet [117].

Similarly, VE-821, another ATR-inhibitor, has been found to radiosensitize HPV-negative HNSCC cell lines in vitro [118,119]. Faulhaber et al. extended these features by testing multiple kinase inhibitors in various cancer cell lines. Among them, AZD0156 (inhibitor of ATM) and VE-822 (ATR inhibitor) both demonstrated superior efficacy compared to radiotherapy alone, with a synergistic effect observed when used in combination with RT [120,121]. In that respect, VE-822 is currently being studied in phase I clinical trials in HNSCC (NCT02567422, NCT03641313), with both trials reporting tolerable toxicity profiles [122,123].

Following DNA damage generation, cells also activate Wee1 kinase, enabling a cell cycle arrest by phosphorylating and inactivating CDK1 [124]. Therefore, targeting Wee1 could be a promising strategy to overcome chemoresistance by preventing cell cycle arrest. Indeed, the Wee 1 inhibitor MK-1775/AZD1775 has shown to increase sensitivity to cisplatin in P53 mutant HNSCC both in vitro and in vivo [125]. Since upregulation of Wee1 was reported in cisplatin-resistant HNSCC, AZD1775 has been explored as a drug to overcome cisplatin resistance [126]. Although the radiosensitizing effect of AZD1775 has not been studied in HNSCC, the drug has shown radiosensitization effects in glioblastoma and pontin glioma cells [127,128]. It is being studied in phase I clinical trials in HNSCC as a monotherapy (NCT 01748825), and in combination with cisplatin (NCT 03028766), cisplatin and radiotherapy (NCT 02585973), and cisplatin + docetaxel (NCT 02508246) [129,130,131,132].

Another DNA repair inhibitor, AZD7762, was developed to target Chk1 or Chk2, preventing the phosphorylation of Cdc25a and Cdc25c, leading to cell cycle progression [133]. In combination with cisplatin, AZD7762 increases cell death in cisplatin-resistant cells with mutated p53 [134]. Although its efficacy in radioresistant cell lines has not yet been investigated in HNSCC, inhibition of Chk1 has shown increased radiosensitivity in p53 mutant cells [135]. Besides AZD7762, other compounds such as CCT24474 and SAR-020106 have been identified as Chk1 inhibitors that can potentially overcome chemoradioresistance in HNSCC. Both molecules have demonstrated a radiosensitizing effect in vitro and in vivo [136,137]. Notably, SAR-020106 was able to radiosensitize p53-deficient, but not p53-wild type cell lines.

Finally, the small molecule prexasertib (LY2606368) is another promising drug that increases cisplatin toxicity and radiosensitizes HNSCC when used in combination with cisplatin and RT, though not when used alone [138]. The combination of prexasertib, cisplatin and radiotherapy was shown to be most effective in vivo. Prexasertib has shown promising results as a monotherapy in the clinical trial NCT 01115790 [139,140]. However, another clinical trial (NCT 02555644) failed to report results in HNSCC patients treated with prexasertib and cisplatin. A similar trial in patients with metastatic colorectal and breast cancer combined prexasertib with various standard of care treatments, which were well tolerated (NCT 02124148) [141,142,143].

Repairing DNA damage via nucleotide excision repair (NER) is one of the main strategies used by chemoresistant HNSCC to survive [47,144]. This pathway is regulated by two cullin-RING ligases (CRLs), CUL4A and CUL4B, which require the conjugation of the ubiquitin-like protein NEDD8 [145]. This process is known as NEDDylation and plays an important role in cellular homeostasis. Pevonedistat (MLN4924), an inhibitor of NEDD8-activating enzyme (NAE) [146], has been shown to disrupt this pathway, increasing DNA damage and cisplatin efficacy in vitro. When combined with cisplatin, pevonedistat induced tumor regression in vivo [147] and increase the sensitivity of HNSCC cells to cisplatin by downregulating DDB2, a downstream target of CUL4A that interacts with DNA lesions [148]. Moreover, pevonedistat has been shown to radiosensitize HNSCC in vitro and to synergize with RT in vivo [149].

Another strategy is to target the MRE11-RAD50-NBS1 (MRN) complex, which is involved in the repair of double-strand breaks (DSB) through HR or NHEJ [150]. The RAD50 component of the complex, which stabilizes DNA ends during repair and maintains telomers, has been recently targeted in different studies [151,152,153,154]. By transfecting HNSCC cells with an adenoviral vector containing a mutated *RAD50* gene (ad-RAD50), researchers observed a decrease in cell proliferation, which was further enhanced when combined with cisplatin both in vitro and in vivo [154]. Additionally, ad-RAD50 as a monotherapy or in combination with cisplatin increased DNA DSB. Similarly, the NBS1 component of the MRN complex has also been targeted using an adenoviral particle (ad-NBS1) [155]. NBS1 is crucial to recognize DNA damage and recruit the other components of the MRN complex. The associated target agent, ad-NBS1, was found to sensitize HNSCC to cisplatin both in vitro and in vivo. Moreover, mutant NBS1 demonstrated a radiosensitization effect in HNSCC, highlighting its potential as a therapeutic target [156].

Overexpression of endothelial growth factor receptor (EGFR) is associated with radioresistance in cancer cells and a poor prognosis in HNSCC [157,158]. Although monoclonal antibodies against EGFR, such as cetuximab, have been developed, their efficacy as monotherapies has been disappointing [159]. EGFR is known to promote DNA double-strand break repair through HR and NHEJ, potentially by activating the MAPK pathway in cancer cells [160]. Consequently, cetuximab monotherapy has been shown to radiosensitize HNSCC via EGFR inhibition [161]. Inhibition of the MAPK-pathway abolished DSB repair, suggesting the involvement of the MAPK-pathway in EGFR mediated DNA repair [162]. Interestingly, sorafenib, an inhibitor of Raf (a component of the MAPK pathway), has been shown to radiosensitize HNSCC, further supporting the role of MAPK in this process [163]. Although sorafenib has been FDA-approved and shown to be well tolerated in phase I clinical trials for recurrent or metastatic HNSCC (NCT00096512, NCT00199160), no clinical trials investigating sorafenib in combination with RT is ongoing [164,165]. It has to be noted that overexpression of EGFR also leads to an overactivation of signal transducer and activator of transcription 3 (STAT3), a key transcription factor involved in various cellular processes including oncogenesis in HNSCC [166,167]. The increase in STAT3 expression has also been associated with radio- and chemoresistance in other cancers [168]. Linifanib (ABT-869), a receptor tyrosine kinase inhibitor, has been shown to inhibit the STAT pathway in acute myeloid leukemia [169]. Hsu et al. demonstrated that linifanib increase the sensitivity of HNSCC cells to radiation by inhibiting STAT3 and its downstream pathways [170].

### 3.2. Targeting Hypoxia

Hypoxia in tumors is a master regulator of RT response, leading to poor prognosis and a reduced treatment efficacy [171]. Reoxygenation before irradiation has been shown to restore radiosensitivity [172]. However, despite hypoxia being an important mechanism for radioresistance, little research has been done with the aim of overcoming radioresistance by targeting hypoxia.

One key player in hypoxia is hypoxia-inducible factor 1-alpha (HIF-1α), which allows cancer cells to survive in a low-oxygen environment and is associated with radioresistance [173]. Therefore, various molecular agents able to target HIF-1α have been explored as a potential radiosensitization strategy, including melittin. By inhibiting the expression of HIF-1α and its signaling [174,175], it has demonstrated radiosensitization in both in vitro and in vivo studies [176].

In addition, certain treatments have been developed to exploit the lower oxygen concentrations of hypoxic cells. One such approach involves CP-506, a hypoxia-activated prodrug, which becomes irreversibly reduced in the absence of oxygen. Once activated in its reduced form, CP-506 induces cytotoxicity by promoting DNA crosslinks. In vivo evaluation of CP-506 in combination with hypofractionated radiotherapy increased locoregional control by 62% and 27%, respectively, for two separate cell lines [177].

### 3.3. Targeting Immune Checkpoints

The use of immunomodulator agents in cancer treatment has become an established part of the therapeutic arsenal, with, for example, the approval of nivolumab for clinical use in HNSCC [178]. It targets the programmed cell death receptor 1 (PD-1) found on the surface of T-cells. When the PD-1 receptor binds to its ligand (PD-L1), T-cells become inactivated and can undergo apoptosis [179]. Interestingly, PD-L1 might carry out other functions and is upregulated in chemoresistant HNSCC cell lines [180,181]. Shen et al. found PD-L1 to be associated with the MRN complex component NBS1. They demonstrated that downregulation of PD-L1 alone or in combination with NBS1 downregulation using siRNA could resensitize cisplatin-resistant HNSCC cells [181]. As PD-L1 can translocated from the cell surface to the nucleus, the use of anti-PD-L1 monoclonal antibodies might be less effective, whereas targeting PD-L1 in other ways might be promising. Several studies, such as the JAVELIN HNC [182] and KEYNOTE-412 [183] studies, have tested combinations of avelumab and pembrolizumab with CRT, respectively. While these trials did not have the desired outcome, certain patients did benefit from this combination, indicating that further stratification of the patients receiving these combinations could be required to observe its full potential.

### 3.4. Targeting Autophagy Pathway

Autophagy is a stress-induced process used by cells to protect themselves. Irradiation is one such trigger of autophagy, leading to the survival of the cell and radioresistance [184]. Microtubule-associated protein 1A/1B-light chain 3 (LC3) is a key protein involved in the process of autophagy and is associated with autolysosomes and autophagosomes [185]. Research has shown that targeting LC3 by transfecting HNSCC with siLC3 can re-sensitize radioresistant HNSCC [186].

### 3.5. Targeting Apoptosis Pathway

As inhibition of apoptosis leads to chemoradioresistance, reactivating the apoptosis pathway may be a promising approach to overcome acquired resistance. Survivin, an inhibitor of apoptosis (IAP), is upregulated in cisplatin-resistant cells [187,188]. YM155, a small molecule that suppresses the expression of survivin, has been shown to reverse cisplatin resistance in HNSCC [187,189]. Additionally, YM155 increases the efficacy of cisplatin both in vitro and in vivo and inhibits tumor growth. In addition to survivin, other IAPs, such as cIAP-1 and cIAP-2, suppress the extrinsic apoptosis pathway, while others like the X-linked inhibitor of apoptosis (XIAP) suppresses the intrinsic apoptosis pathway [190]. These proteins are inhibited by the second mitochondria-derived activator of caspase (SMAC), allowing apoptosis to proceed. Induction of ubiquitination and degradation of cIAP-1 by SMAC mimetics are a feasible treatment [191,192,193]. SMAC mimetic SM-164 has been shown to radiosensitize HNSCC both in vitro and in vivo [194]. Furthermore, studies on the knockdown of Bcl-2 have shown that siRNA targeting Bcl-2 can radiosensitize HNSCC [13,195]. XEVINAPANT, an IAP inhibitor, has been shown in a phase II clinical trial to improve local control when combined with CRT and is further being studied in a phase III trial [196].

### 3.6. Oxidative Stress

Upregulation of *PDK2* is associated with drug resistance in various cancers [197,198] including HNSCC [199]. Pyruvate, a natural PDK2 inhibitor, and its structural analog dichloroacetate (DCA) have proven effective in shifting the energy production from aerobic glycolysis to mitochondrial oxidative phosphorylation [200,201]. This triggers the reactivation of PDC, the TCA cycle and mitochondrial glucose oxidation. Roh et al. confirmed the association between *PDK2* upregulation and cisplatin resistance in HNSCC. Their study demonstrated that treating HNSCC with DCA resensitized the cells to cisplatin both in vitro and in vivo but also induced ROS accumulation [202].

Nuclear factor erythroid 2-related factor 2 (NRF2) is involved in the response to oxidative stress and is involved in chemo- and radioresistance when overexpressed [203]. Targeting NRF2 using siRNA resensitizes the cells to both radiotherapy and cisplatin [204,205]. Beyond siRNA, the flavonoid wogonin has been shown to suppress NRF2-mediated cellular defense responses and to induce ROS overproduction [206,207,208]. In cisplatin-resistant HNSCC, wogonin can selectively induce ROS accumulation and GSH depletion, resulting in a resensitization of the cells to cisplatin [209].

The triterpenoid hederagenin has previously been shown to be cytotoxic in various types of cancer [210]. Hederagenin’s toxicity can be ascribed to multiple mechanism of action, including interference with the NRF2 pathway, which leads to cell death in cisplatin-resistant HNSCC in vitro and inhibits growth in vivo [211]. Additionally, it has been proposed to activate components of the intrinsic apoptosis pathway and inhibit late-phase autophagy in various cancers [212,213].

While the previously mentioned compounds primarily address cisplatin resistance, 4-methylumbelliferone (4-MU) offers a broader approach by also radiosensitizing HNSCC. 4-MU inhibits the synthesis of hyaluronic acid and is demonstrated to be effective as monotherapy or in combination with radiotherapy in both radiosensitive and radioresistant HNSCC [214]. Hyaluronic acid is a ligand of CD44, a receptor which plays various roles in cancer cell survival. By inhibiting CD44 ligand synthesis, 4-MU reduces the resistance to oxidative stress, as evidenced by increased ROS levels and decreased superoxide dismutase production [214]. It is currently being investigated in clinical trials for primary sclerosing cholangitis (NCT05295680), COVID-19 (NCT 05386420) and pulmonary hypertension (NCT05128929) under the names hymecromone, cantabiline and/or isochol. Additionally, inhibiting CD44 itself with 1,2,3,4 tetrahydroisoquinoline (THIQ) has been shown to sensitize cells to cisplatin through different pathways [215,216].

### 3.7. Others

Focusing on the main resistance mechanism is one approach to overcoming chemoradioresistance. However, some studies have identified other intriguing methods for resensitizing HNSCC, which do not directly target these primary resistance pathways.

One of such approaches involves the use of the chicken anaemia viral protein apoptin, which can selectively accumulate in cancer cells including HNSCC [217]. Apoptin was shown to be effective as a monotherapy in radiosensitive and -resistant HNSCC as well as in combination with RT in vitro [218]. Despite these encouraging results, an in vivo study in dogs revealed only partial oncolysis [219], indicating that further research is necessary to fully explore apoptin’s potential as a therapeutic agent in HNSCC.

Another unconventional target for overcoming chemoradioresistance is the vitamin D receptor. In the kidneys, vitamin D is converted into its active form, calcitriol, which binds to the vitamin D receptor. Once bound, the receptor translocates to the nucleus, interacts with the retinoid X receptor and regulates the transcription of specific DNA segments [220]. Khamis et al. reported an association between vitamin D receptor overexpression and cisplatin-resistance in HNSCC [221], suggesting that the vitamin D receptor has a ligand-independent effect on cisplatin resistance [222,223]. However, pre-incubation of cells in presence of calcitriol or an analog can overcome this cisplatin resistance, pointing to a ligand-dependent effect as well. In this context, the calcitriol analog maxacalcitol may be a potential treatment for cisplatin-resistant HNSCC [221].

Lastly, histone deacetylase 6 (HDAC6) represents a novel interesting target since it is upregulated in cisplatin-resistant HNSCC [224]. Deacetylation of histones leads to the condensation of chromatin, preventing genes from being transcribed. HDAC6 also has non-histone targets and both functions are involved in cancer development [225]. Tavares et al. studied the role of HDAC6 in cisplatin-resistant HNSCC by inhibiting HDAC6 with tubastatin A. They reported that HDAC6 was increased in cisplatin-resistant HNSCC and that treatment with tubastatin A overcame cisplatin resistance, in monotherapy and in combination with cisplatin [224].

## 4. Generating Acquired Resistance In Vitro

Besides using tissue samples from patients presenting with resistance, one could also establish chemo- and/or radiotherapy-resistant cell lines. Below, we summarize some frequently used protocols.

### 4.1. Acquired Resistance to Cisplatin

To generate cisplatin-resistant cell lines, exposing the cells to increasing concentrations of cisplatin is the most common approach. Unfortunately, there seems to be no consensus on concentrations of cisplatin to which cells should be exposed. Some research groups use dose-escalation schemes in which cells are exposed to initial concentrations of CDDP of 1 µM and resistant clones are selected by gradually increasing the concentration to 25–50 µM [187,226,227,228]. The choice of concentration seems to be arbitrary, as research groups who use the same cell line decided to use different concentrations. Alternatively, other groups generate cisplatin-resistant cells by daily culturing them in cisplatin-containing medium without increasing concentrations across time [106,107,108,109].

The time it takes to establish the cisplatin-resistant cell lines differs from 6 months [19,187,229,230,231] to 15 months [227], depending on the research group. It is worth noting that not all research groups report the total time required for cell line establishment.

The viability and the proliferation of the established cell lines were tested by performing an MTT assay [19,187,228,229] or a BrdU assay [228], respectively, and compared with the original cell lines.

### 4.2. Acquired Resistance to Radiotherapy

When it comes to establishing radioresistant cell lines, research groups tend to go for a protocol that is similar to the radiotherapy treatment given to a patient. Indeed, protocols mention fractionated doses and total doses of 60–120 Gy. However, the fractionated dose and the time taken to establish the cells tend to be different. The most commonly used fractionated dose schedule is 2 Gy/fraction [232,233,234,235,236], even though hypofractionnated regimens of 5 and 10 Gy were also reported [237,238].

These repeated irradiations are carried out over long periods, ranging from 6 [232,236] to 42 weeks [238], with recovery periods between the fractionated doses ranging between a day [232,236] and several weeks [238]. Besides irradiating at specific time points, allowing cells to reach a certain confluence is also a frequent strategy. This results in cultures derived from the surviving fraction of the previously irradiated culture, selecting for cells that are most resistant. Continually irradiating without regard for confluence will result in cultures dying out, being unable to proliferate. Fukuda et al. and Song et al. irradiated cells and waited until the cells reached 90% and 80% confluency, respectively [233,235].

To test the acquired radioresistance of the cell lines, clonogenic assays [232,233,234,235,236,237,238,239] are often used.

### 4.3. Acquired Resistance to CRT

Few groups have studied resistance to CRT and only one research group has reported successful generation of cell lines resistant to both cisplatin and radiotherapy. Hagege et al. studied the polo-like kinase 1 (Plk1) inhibitor onvansertib [240]. Plk1 is a cell cycle regulator that is overexpressed in HNSCC. To generate the cisplatin-resistant cell lines, human HNSCC cells (CAL27 and CAL33) were exposed to increasing concentrations of cisplatin until a maximum concentration of 10 µM was reached. The radioresistant cell line was established by irradiating the cells at 8 Gy for 25 cycles. Finally, CRT resistance was achieved by irradiating the cisplatin-resistant cell lines in the same 25 cycles of 8Gy conditions. A cell viability assay and a clonogenic assay confirmed the chemo-radioresistance of the cell lines.

## 5. Conclusions

In HNSCC, both intrinsic and acquired resistance to CRT are major challenges, as more than half of HNSCC patients experience relapse despite intensive CRT. This resistance in HNSCC can result from multifaceted reasons, including DNA/RNA damage repair, drug efflux, apoptosis inhibition and the presence of cancer stem cells (CSCs) with high expression of stemness-related markers, etc. To overcome these resistance mechanisms, precision medicine approaches and combination treatment strategies are being explored. These may include targeting specific molecular mechanisms of resistance and personalizing treatment strategies for HNSCC patients. Overall, understanding the molecular mechanisms of CRT resistance and developing targeted approaches are crucial for improving the treatment outcomes and increasing the survival of HNSCC patients. 

## Data Availability

No new data were created or analyzed in this study.
